# Humoral responses in naive or SARS-CoV-2 experienced individuals vaccinated with an inactivated vaccine

**DOI:** 10.1038/s41421-021-00311-z

**Published:** 2021-08-17

**Authors:** Pai Peng, Hai-jun Deng, Jie Hu, Xiao-yu Wei, Jian-jiang Xue, Ting-ting Li, Liang Fang, Bei-zhong Liu, Ai-shun Jin, Feng-li Xu, Kang Wu, Quan-xin Long, Juan Chen, Kai Wang, Ni Tang, Ai-long Huang

**Affiliations:** 1 Key Laboratory of Molecular Biology for Infectious Diseases (Ministry of Education), Institute for Viral Hepatitis, Department of Infectious Diseases, the Second Affiliated Hospital, Chongqing Medical University, Chongqing, China; 2 Yong-Chuan Hospital, Chongqing Medical University, Chongqing, China; 3 University-Town Hospital of Chongqing Medical University, Chongqing, China; 4 Department of Immunology, College of Basic Medicine, Chongqing Medical University, Chongqing, China

**Keywords:** Immunology, Cell biology

Dear Editor,

The humoral immune response to severe acute respiratory syndrome coronavirus 2 (SARS-CoV-2) is critical for the clearance of the virus and also plays a key role for the prevention of viral reinfection. It has been extensively reported that antibody response to SARS-CoV-2 tends to be diminished in course of time^1–3^. Thus, the durability of the protective immune response in coronavirus disease-2019 (COVID-19) recovered patients is of great interest. There is increasing appreciation of the key role that immunological memory plays in durable protective immunity after infections or vaccinations, even with lower antibody titers^4,5^. Inactivated vaccines as a conventional vaccine development have been shown to be effective among other viruses^6^. It has raised concern about the impact of prior infection by SARS-CoV-2 on the immune response induced by inactivated vaccines. For these reasons, we examined the humoral immunity in convalescent patients for 12 months postsymptom onset (PSO) and evaluated the immune response elicited by an inactivated vaccine in naive or COVID-19 recovered individuals.

170 blood samples from a follow-up cohort of 85 COVID-19 patients were collected over a 12-month period PSO (Supplementary Fig. S1a). Participants with 57.6% male and 42.4% female aged from 3 to 84 (median: 48 years) were enrolled (Supplementary Table S1). After the measurement of neutralizing antibodies (NAbs), five participants with low NAb titers were given two injections of CoronaVac vaccine (developed by Sinovac Life Sciences, China) 21 days apart for the study of immunological memory response. Meanwhile, 19 healthy individuals were recruited as the control group (Supplementary Fig. S1b, Table S2).

Anti-SARS-CoV-2 spike (anti-S) IgG/IgM/IgA and NAb titers were measured with previously described MCLIA kits and pseudovirus-based neutralization assay. Anti-S IgG and NAbs were still detectable in 95.5% (42 of 44) and 93.2% (41 of 44) serum samples, respectively, at 12 months PSO (Fig. 1a). Correlation between anti-S IgG levels and Nab titers (*r* = 0.64, *p* = 5.8e−21) was shown over the study period (Supplementary Fig. S2a). Nevertheless, during the 12-month follow-up visit in the COVID-19 recovery cohort, anti-S IgG/IgM/IgA and NAb titers represented a sustained decline (Fig. 1a, Supplementary Fig. S2b, c). For the neutralizing antibodies, median of NAb titers decreased from 631 at Month 1 to 604 at Month 3, to 134 at Month 8 and to 84 at Month 12. For the IgG antibodies, the median of signal-to-cutoff ratio (S/CO) dropped from 28.6 at Month 1 to 27.7 at Month 3, 11.5 at Month 8 and 7.2 at Month 12. At Month 12, the levels of specific antibodies were much lower than the levels at Month 1 (82.8%, 96.4%, and 89.4% decrease for IgG, IgM, and IgA antibodies, respectively). In addition, a longitudinal study was observed among nine participants provided samples at all follow-up time points. In spite of a general decline in humoral immune response, the dynamic changes showed significant variation between anti-S IgG/IgM/IgA antibodies and NAbs (Supplementary Fig. S2d–g). Both IgM and IgA levels in 7 of 9 individuals reached peak at 1 month PSO and fell below the positive threshold thereafter. By contrast, IgG and NAbs decreased slowly and remains 100% (9/9) and 78% (7/9) positive at 12 months PSO.

**Fig. 1:**
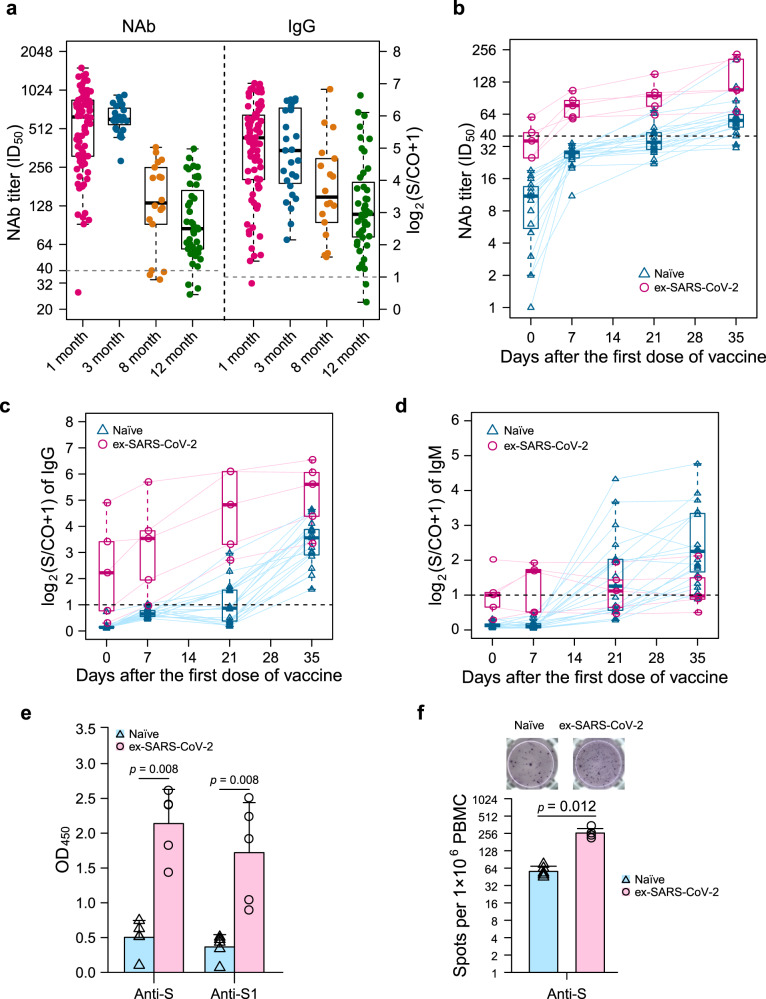
Immunological memory response of COVID-19 recovered individuals elicited by an inactivated vaccine at 12 months PSO. **a** Dynamic changes of antibody response in a cohort of COVID-19 recovered individuals from 1 to 12 months. SARS-CoV-2 specific IgG/IgM/IgA and NAb titers were measured with previously described MCLIA kits and pseudovirus-based neutralization assay. Medians (interquartile range, IQR) are shown. The NAb titers were calculated as 50% inhibitory dose (ID_50_) and the limit of detection (LOD) was 40; the signal to cut-off ratio (S/CO) of IgG/IgM/IgA above 1 was considered as positive. NAb titers (**b**), IgG (**c**), and IgM (**d**) levels of two cohort in which COVID-19 convalescent individuals or healthy participants were injected by two-dose inactivated vaccine CoronaVac; **e**, **f** the status of SARS-CoV-2 specific memory B cells in COVID-19 recovered individuals and naive individuals. Enzyme-linked immunosorbent assay (ELISA) (**e**) was performed to detected anti-S, anti-S1 IgG secreted by memory B cells and enzyme-linked immunosorbent spot assay (ELISpot) (**f**) was performed to analyze the number of antibody-secreting cells. OD denotes optical density, S spike protein and S1 fragment of spike glycoprotein. Empty triangles with red and empty circles with blue indicate healthy individuals and SARS-CoV-2 experienced individuals, respectively; the horizontal dashed lines denote the lower LOD. In **a**–**d**, boxes denote the median, first and third quartiles, while the whiskers show ×1·5 interquartile range (IQR) of antibody levels. In **e**, **f**, boxes and error bars denote mean ± standard deviation. Statistical analysis was performed with the use of the two-tailed, nonparametric Mann–Whitney *U* test.

Blood samples from two vaccination cohorts were collected pre-vaccination (day 0, the day before the first dose of vaccine) and 7, 21, 35 days after the first dose of vaccine (Supplementary Fig. S1b). The evaluation of immunological memory induced by the inactivated vaccine was performed by detection of specific antibodies and antibody-secreting memory B cells among participants. NAbs were detective only in COVID-19 recovered group within 7 days after the first dose of vaccine (median of NAb titers 36 on Day 0; 77 on Day 7; 95 on Day 21; and 108 on Day 35) (Fig. 1b). The median NAbs titer was 56 in the naive group 35 days after the first dose of vaccine. Due to the previous presence of SARS-CoV-2 specific antibodies, the majority of COVID-19 recovered individuals had detectable IgG from pre-vaccination to post-vaccination (median S/CO value before vaccination, 3.68; and 10.59, 27.33, and 47.74 on Day 7, 21, and 35 after vaccination, respectively) (Fig. 1c). In the naive group, anti-S IgG was detected with lower values than COVID-19 recovered individuals over 35 days after the first dose of vaccine (median S/CO value before vaccination, 0.10; and 0.57, 0.83, and 10.81 on Day 7, 21, and 35 after the first dose of vaccine, respectively). IgG levels of COVID-19 recovered individuals were 4.4 times that of naive individuals at Day 35 (median S/CO value, 47.74 vs 10.81). Interestingly, IgM titers increased over time in naive group, while no substantial changes displayed in COVID-19 recovered group (Fig. 1d). Furthermore, IgA of both groups remained at a low level, even staying below the positive threshold (Supplementary Fig. S3).

To further understand higher humoral response in COVID-19 recovered individuals after vaccination, SARS-CoV-2 specific memory B cells differentiated from peripheral blood mononuclear cells of 5 SARS-CoV-2 experienced and naive individuals before vaccination were determined by enzyme-linked immunosorbent assay (ELISA) and enzyme-linked immunosorbent spot assay (ELISpot). As expected, specific anti-S, anti-S1 fragment of spike glycoprotein (anti-S1) IgG and the number of anti-S IgG antibody-secreting cells presented higher levels in SARS-CoV-2 experienced group than the naive group (Fig. 1e, f).

Our findings demonstrated that anti-S IgG, IgM, IgA and NAb titers declined gradually over 1 year in patients infected with SARS-CoV-2. Even though antibody response of most participants remained detectable, the drop of more than 80% were shown in anti-S IgG, IgM, IgA, and NAb titers. To evaluate the duration of protective immunity against SARS-CoV-2, further surveillance is needed. Moreover, our results suggest that immunological memory mediated by an inactivated vaccine could recall higher response of IgG and NAb in COVID-19 recovered individuals with low NAb titers than in naive persons at 12 months PSO. After infection, SARS-CoV-2 specific memory B cells secreting antibody increased significantly in COVID-19 recovered individuals compared to healthy controls. It should be pointed out that maybe due to the cross-activity between SARS-CoV-2 and seasonal coronaviruses, SARS-CoV-2 S and S1-specific antibodies secreted by memory B cells were detected at baseline in naive persons^7^.

Compared to our data, rapid immune response elicited by a single mRNA vaccine dose was showed in several SARS-CoV-2 recovery cohorts vaccinated by mRNA-based vaccines^8–11^. Further investigation is needed to answer the necessity of vaccination for SARS-CoV-2 experienced individuals, and to answer whether the immune response provides effective protection from reinfection in this special group, especially for SARS-CoV-2 variants.

The main limitation of this study is the small sample size and relatively short period for the observation of vaccination cohorts. Even though our data provided a hint about the role of memory B cell response in humoral response after vaccination or reinfection, a deeper investigation carried out by flow cytometry will be needed. An inactivated virus vaccine including all components of SARS-CoV-2 might provide the distinct benefit to boost T-cell response against other SARS-CoV-2 proteins, but T-cell immunity was not investigated in our study.

Our results reveal the durability of immunological response 1 year after natural SARS-CoV-2 infection and the benefit from inactivated vaccines for COVID-19 recovered individuals. It provides more information about immunological characteristics of SARS-CoV-2 inactivated vaccines, thus will contribute to the development of vaccines and the new strategies of vaccination.

## Supplementary information

Supplementary Figures and Tables

## References

[1] Long Q-X (2020). Antibody responses to SARS-CoV-2 in patients with COVID-19. Nat. Med..

[2] Wang, K. et al. Longitudinal dynamics of the neutralizing antibody response to SARS-CoV-2 infection. *Clin. Infect. Dis*. 10.1093/cid/ciaa1143 (2020). 10.1093/cid/ciaa1143 PMC7454328 32745196

[3] Lau EHY (2021). Neutralizing antibody titres in SARS-CoV-2 infections. Nat. Commun..

[4] Gaebler C (2021). Evolution of antibody immunity to SARS-CoV-2. Nature.

[5] Dan JM (2021). Immunological memory to SARS-CoV-2 assessed for up to 8 months after infection. Science.

[6] Jackson ML (2017). Influenza vaccine effectiveness in the United States during the 2015-2016 season. N. Engl. J. Med..

[7] Song G (2021). Cross-reactive serum and memory B-cell responses to spike protein in SARS-CoV-2 and endemic coronavirus infection. Nat. Commun..

[8] Krammer F (2021). Antibody responses in seropositive persons after a single dose of SARS-CoV-2 mRNA vaccine. N. Engl. J. Med..

[9] Ebinger JE (2021). Antibody responses to the BNT162b2 mRNA vaccine in individuals previously infected with SARS-CoV-2. Nat. Med..

[10] Bradley T (2021). Antibody responses after a single dose of SARS-CoV-2 mRNA vaccine. N. Engl. J. Med..

[11] Manisty C (2021). Antibody response to first BNT162b2 dose in previously SARS-CoV-2-infected individuals. Lancet.

