# Supplementary material for: Humoral responses in naive or SARS-CoV-2 experienced individuals vaccinated with an inactivated vaccine
Source: Cell Discov. 2021 Aug 17;7:68. doi: 10.1038/s41421-021-00311-z (PMC8368000; doi:10.1038/s41421-021-00311-z)
Supplement: Supplementary file 1 — Supplementary Figures and Tables [file 41421_2021_311_MOESM1_ESM.pdf]

Immunological responses in naïve or SARS-CoV-2 experienced individuals vaccinated with an inactivated vaccine

Pai Peng[1,#], Hai-jun Deng[1,#], Jie Hu[1,#], Xiao-yu Wei[2], Jian-jiang Xue[3], Ting-ting Li[4], Liang Fang[2], Bei-zhong Liu[2], Ai-shun Jin[4], Feng-li Xu[1], Kang Wu[1], Quan-xin Long[1], Juan Chen[1], Kai Wang[1,*], Ni Tang[1,*], Ai-long Huang[1,*]

Author Affiliations:

[1]Key Laboratory of Molecular Biology for Infectious Diseases (Ministry of Education), Institute for Viral Hepatitis, Department of Infectious Diseases, the Second Affiliated Hospital, Chongqing Medical University, Chongqing, China.

[2]Yong-Chuan Hospital, Chongqing Medical University, Chongqing, China.

[3]University-Town Hospital of Chongqing Medical University, Chongqing, China.

[4]Department of Immunology, College of Basic Medicine, Chongqing Medical University, Chongqing, China.

[#]These authors contributed equally to this work.

***Corresponding author:** Ai-long Huang (ahuang@cqmu.edu.cn) or Ni Tang (nitang@cqmu.edu.cn) or Kai Wang (wangkai@cqmu.edu.cn).

Key Laboratory of Molecular Biology for Infectious Diseases (Ministry of Education), Institute for Viral Hepatitis, Department of Infectious Diseases, the Second Affiliated

Hospital, Chongqing Medical University, Chongqing, China.

Yixue Yuan Road No.1, Chongqing, 400016, P. R. China

Tel/Fax: 86-23-68486780

Supplementary information

Materials and Methods

Trial design and oversight

To examine the humoral immunity elicited by an inactivated vaccine in convalescent patients of COVID-19, a follow-up study over 12 months post symptom onset (PSO) was performed on 85 subjects who had recovered from SARS-CoV-2 infection in Yongchuan Hospital Affiliated to Chongqing Medical University (Chongqing, China). According to the evaluation of antibody response among these participants at 12 months PSO, 5 convalescents who have low neutralizing antibody (NAb) titers and 19 healthy persons as the control group were recruited for two injections of a COVID-19 inactivated vaccine CoronaVac (Lot no. 20200830) (developed by Sinovac Life Sciences, Beijing, China) (3μg / 0.5 mL per dose, at Day0 and Day21 right after blood sampling).

Determination of IgM, IgG and IgA antibody titers against SARS-CoV-2

The SARS-CoV-2 specific IgG/IgM/IgA antibodies were measured by commercially available Magnetic Chemiluminescence Enzyme Immunoassay Kits (developed by Bioscience Co., Ltd. Tianjin, China) according to the manufacturer’s instructions. Antibody levels were presented as the ratio between the chemiluminescence signal and the cutoff value (S/CO). The test was regarded as positive, when the S/CO value is higher than 1.0.

SARS-CoV-2 pseudotype neutralization assays

The neutralization assays based on pseudovirus particles were performed as previously described. Briefly, SARS-CoV-2 spike pseudotyped virus were obtained in the supernatant of co-transfected HEK293T cells with pNL4-3.Luc.R-E- plasmid and recombinant SARS-CoV-2 S plasmid. 50uL pseudovirus (3.8×10^4 copies) was pre-incubated with serial dilutions of serum samples from participants and human control serum as a negative control in a 1:1 ratio for 1 hour at 37°C and then added to 96-well plates pre-coated with 293T-ACE2 cells (1.5×10^4 cells/well). After 12 hours, the culture medium was refreshed. After 72-hour post infection, the 293T-ACE2 cells were lysed with 30 µL lysis buffer (Promega, Madison, WI) to measure pseudoviral transduction. Relative luminescence units (RLUs) of Luciferase activity in the cell lysate were determined using the Luciferase Assay Kit (Promega). The titers of NAbs were calculated as 50% inhibitory dose (ID_{50}), expressed as the dilution of serum samples that induced a 50% reduction of RLUs compared with virus control.

Assessment of SARS-CoV-2 specific memory B cell response

After the improvement of an established method in our lab (by Long QX et al. Immune memory in convalescent patients with asymptomatic or mild COVID-19. Cell Discov 2021. doi: 10.1038/s41421-021-00250-9), 10ml EDTA-K2 anticoagulant whole blood samples of healthy individuals and COVID-19 patients were collected for the isolation of peripheral blood mononuclear cells (PBMCs) using Lymphoprep™ (STEMCELL Technologies, catalog no. 1114547) gradient centrifugation (10min,

1200×g, RT, with the brake off). 2×10^6 PBMCs were cultured in 24-well plates at 37°C, 5% CO_2 in the presence of 1 μg/mL R848 (Mabtech) and 10 ng/mL IL-2 (Mabtech). After the incubation for 6-8 days, stimulated cells were seeded on ELISpot plates pre-coated recombinant SARS-CoV-2 S protein. After incubation and wash, spot numbers of cells producing specific SARS-CoV-2 Spike IgG antibody (anti-S) were analyzed by recognition of alkaline phosphatase (ALP)-conjugated anti-Human IgG and staining of BCIP/NBT substrate. The supernatant was used to determine specific anti-S and anti-S1 IgG levels of antibody-secreting cells.

Ethical consideration

The study was approved by Chinese clinical test registration center (The world health organization international clinical trials registered organization registered platform, registration number: ChiCTR2100042528). The research protocol was approved by The Ethics Committees of Chongqing Medical University. All participants had provided personal signed informed consent prior to the implementation of this study. Following the operational guide for venous blood collection and COVID-19 vaccination separately issued by National Health Commission of the People's Republic of China and Chinese Center for Disease Control and Prevention, sample collection and vaccination were performed.

Statistical analysis

Data were analyzed using R software (version 3.6.0). Continuous variables were

presented as the median (interquartile range, IQR) or mean ± standard deviation (sd), and the comparison between two groups were evaluated using the two-tailed, non-parametric Mann-Whitney U test. Categorical variables were presented as numbers (%), and the comparison between two groups were analyzed using Fisher's exact test. For the correlation analyses, Pearson correlation was performed. The box plots showed the medians (middle line), first and third qurtiles (boxes) and 1.5x IQR (whiskers), and bar plot showed the mean ± sd. P value less than 0.05 was considered statistically significant.

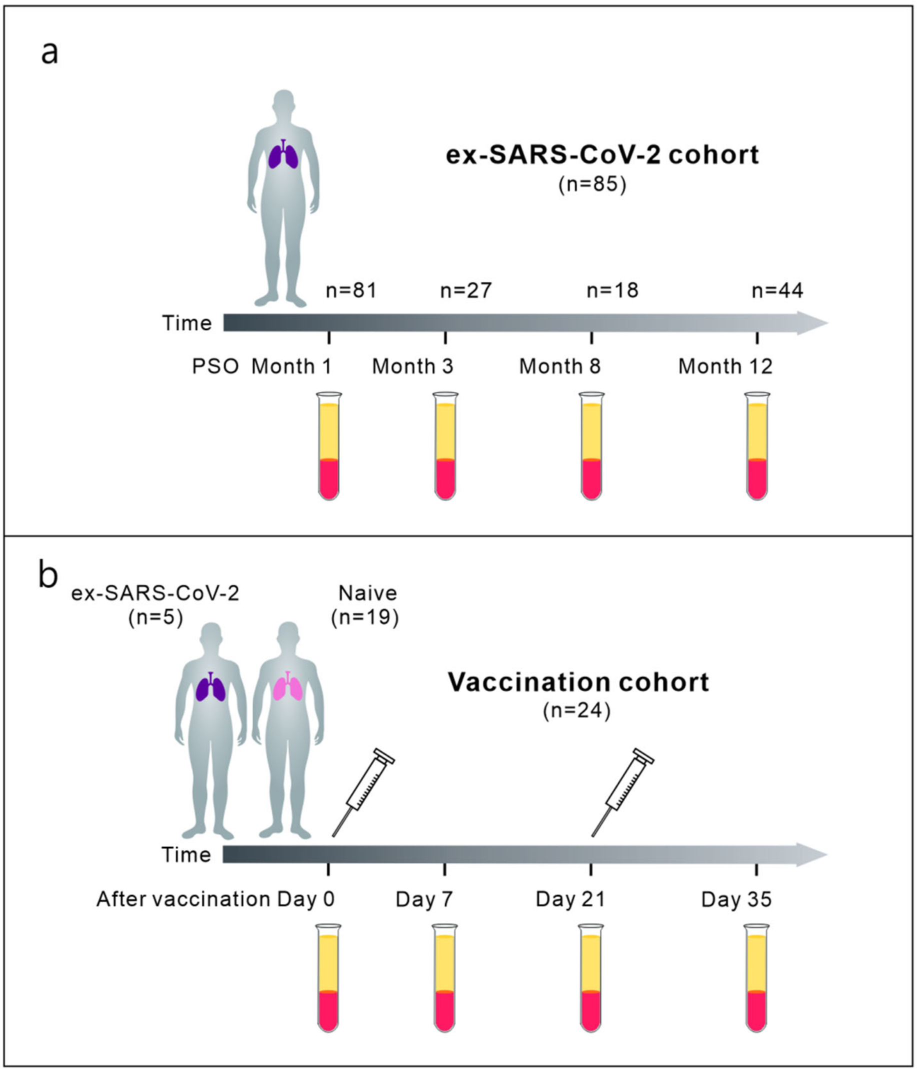

Supplementary Figure S1. Study cohorts and trial design.

Panel A illustrates study timeline of 12-month follow-up visit and number of individuals analyzed at each point of study visit. Panel B represents vaccination program among two cohorts and timepoint of sample collection for participants in days since vaccination.

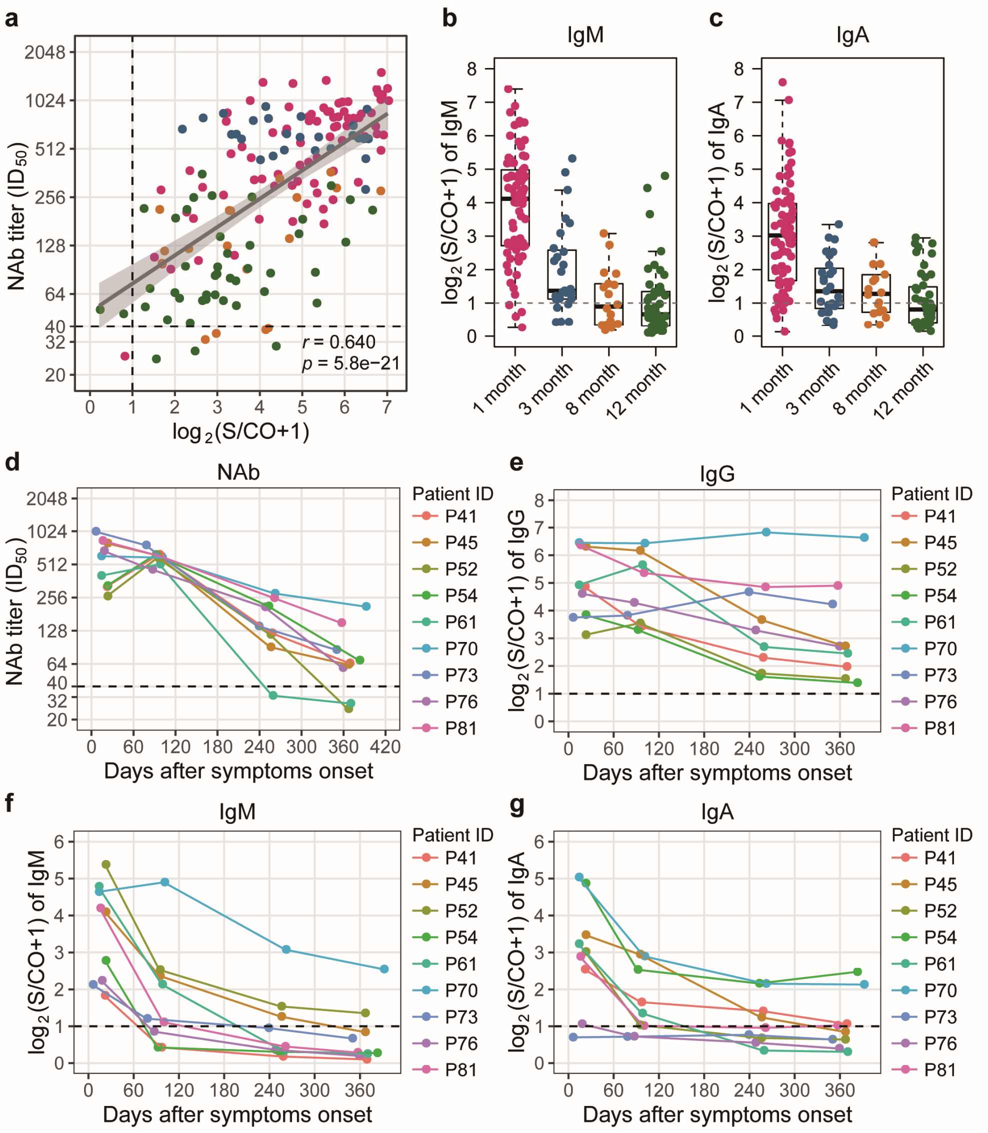

Supplementary Figure S2. Humoral immune response of SARS-CoV-2 recovered individuals over 12-month cohort study.

Shown data are antibody response of SARS-CoV-2 infected participants from Month 1 to Month 12 PSO. Panel A represents the correlation between SARS-CoV-2 specific IgG antibodies and neutralizing antibodies. Panel B and C show IgM and IgA response in COVID-19 convalescent individuals. Pane D-G show a longitudinal

observation of antibody response to SARS-CoV-2 over 12 months.

Pearson correlation was performed for correlation analysis.

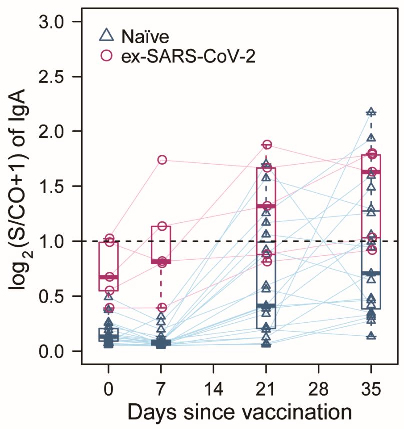

Supplementary Figure S3. IgA antibody level of vaccinated individuals.

Shown is IgA response of participants who were COVID-19 recovered individuals or naïve after 2-dose vaccination. Empty triangles with red and empty circles with blue indicate SARS-CoV-2 experienced individuals and healthy individuals, respectively; the horizontal dashed lines denote the lower LOD. Boxes denote the median, first and third quartiles, while the whiskers show 1.5× interquartile range (IQR) of antibody levels.

Table S1. Clinical characteristics of 85 ex-SARS-CoV-2 individuals enrolled in this study.

Characteristics	Patients (n=85)
**Age (median, IQR)**	48 (40-59)
**Sex (Male/Female)**	
Male	49 (57.6%)
Female	36 (42.4%)
**Exposure**	
From Wuhan	33 (38.8%)
Close contacts	45 (52.9%)
Unknown	7 (8.2%)
**Severity**	
Non-severe	75 (88.2%)
Severe	10 (11.8%)
**Hospitalization days**	16 (12-25)
**Comorbidities**	
Hypertension	15 (17.6%)
Diabetes	8 (9.4%)
Cardiovascular disease	4 (4.7%)
COPD	2 (2.4%)
Chronic liver disease	5 (5.9%)

Chronic kidney disease	1 (1.2%)
Tuberculosis	3 (3.5%)
**Signs and symptoms**	
Fever	41 (48.2%)
Dry cough	36 (42.4%)
Expectoration	21 (24.7%)
Fatigue	20 (23.5%)
Myalgia	12 (14.1%)
Pharyngalgia	12 (14.1%)
Chill	9 (10.6%)
Dypnea	9 (10.6%)
Chest stuffiness	8 (9.4%)
Inappetence	6 (7.1%)
Diarrhea	6 (7.1%)
Nausea	4 (4.7%)
Dizziness	6 (7.1%)
Headache	4 (4.7%)
Vomiting	2 (2.4%)
Rhinorrhea	4 (4.7%)

Table S2. Clinical characteristics of vaccinated individuals enrolled in this study.

	ex-SARS-CoV-2 (n=5)	Naïve (n=19)	*p*
Age (median, IQR)	47 (38-51)	36 (33-43)	0.165
Sex (Male/Female)			1.000
Male	2 (40.0%)	9 (47.4%)	
Female	3 (60.0%)	10 (52.6%)	
BMI, kg/m2	23.9 (21.4-24.2)	24 (22.3-25.8)	0.776
